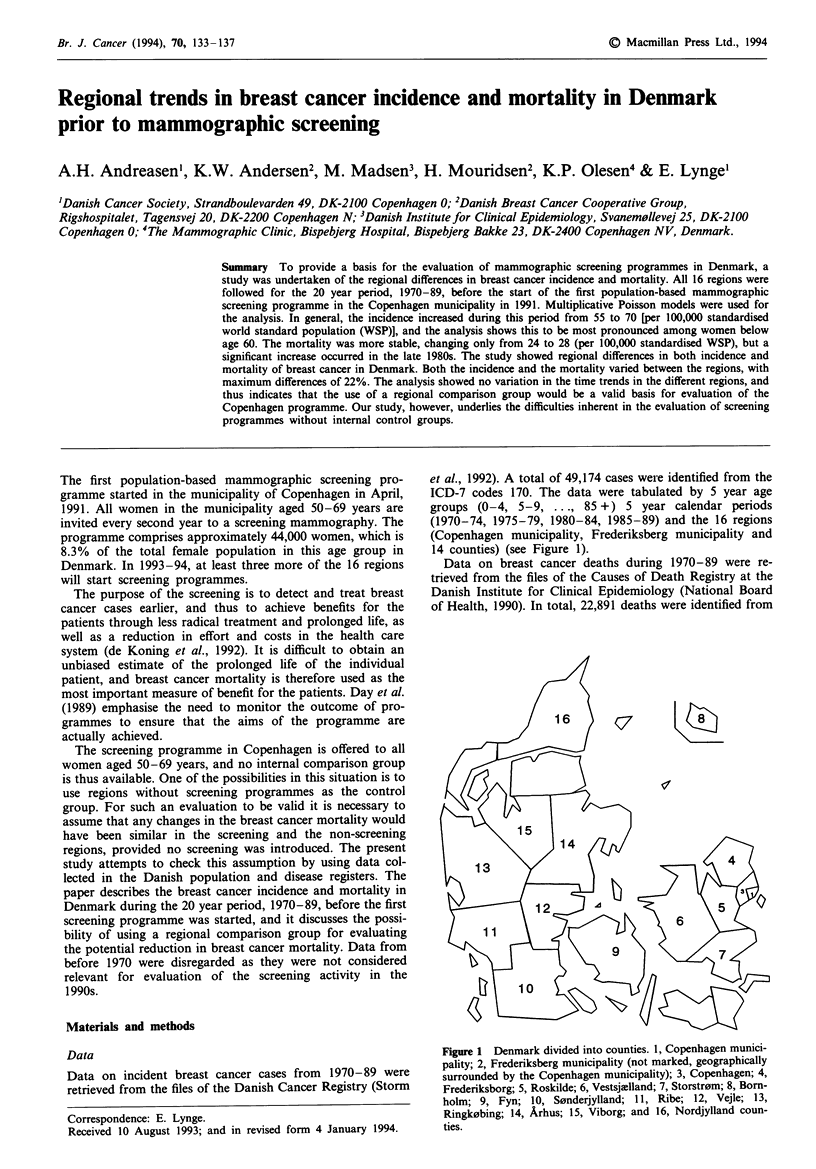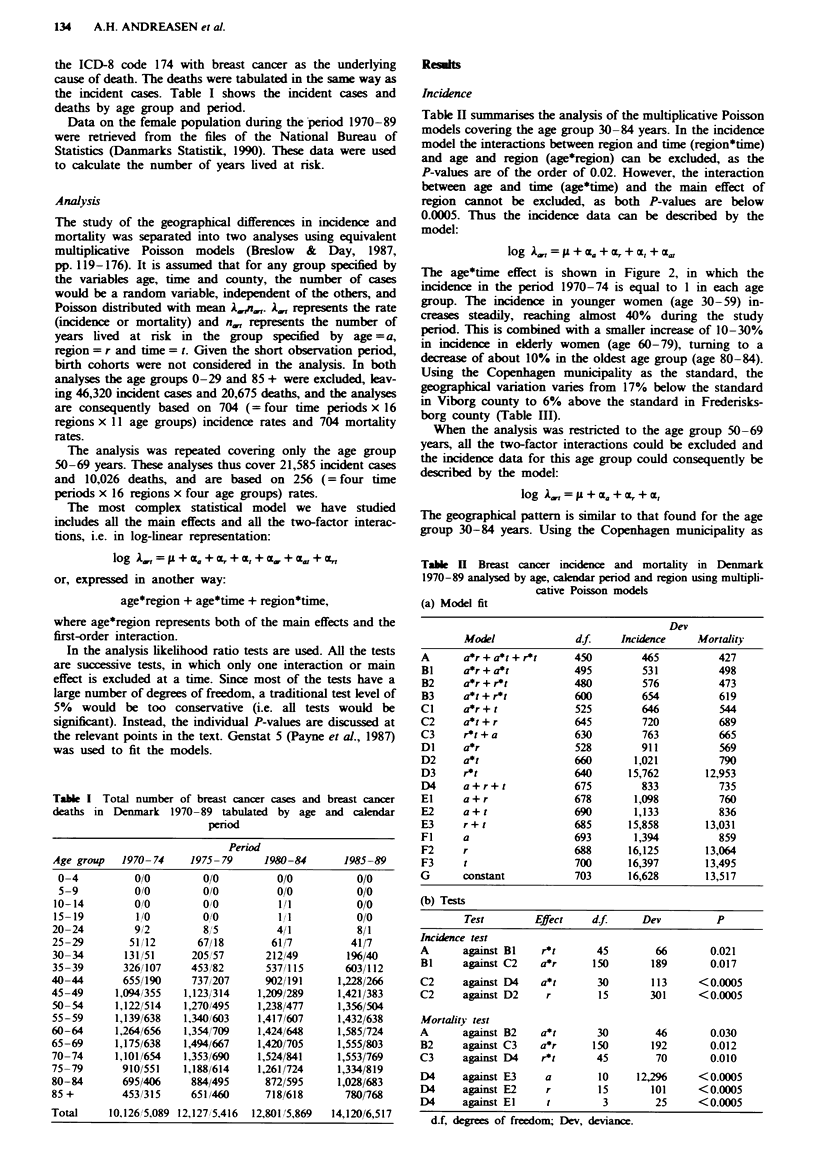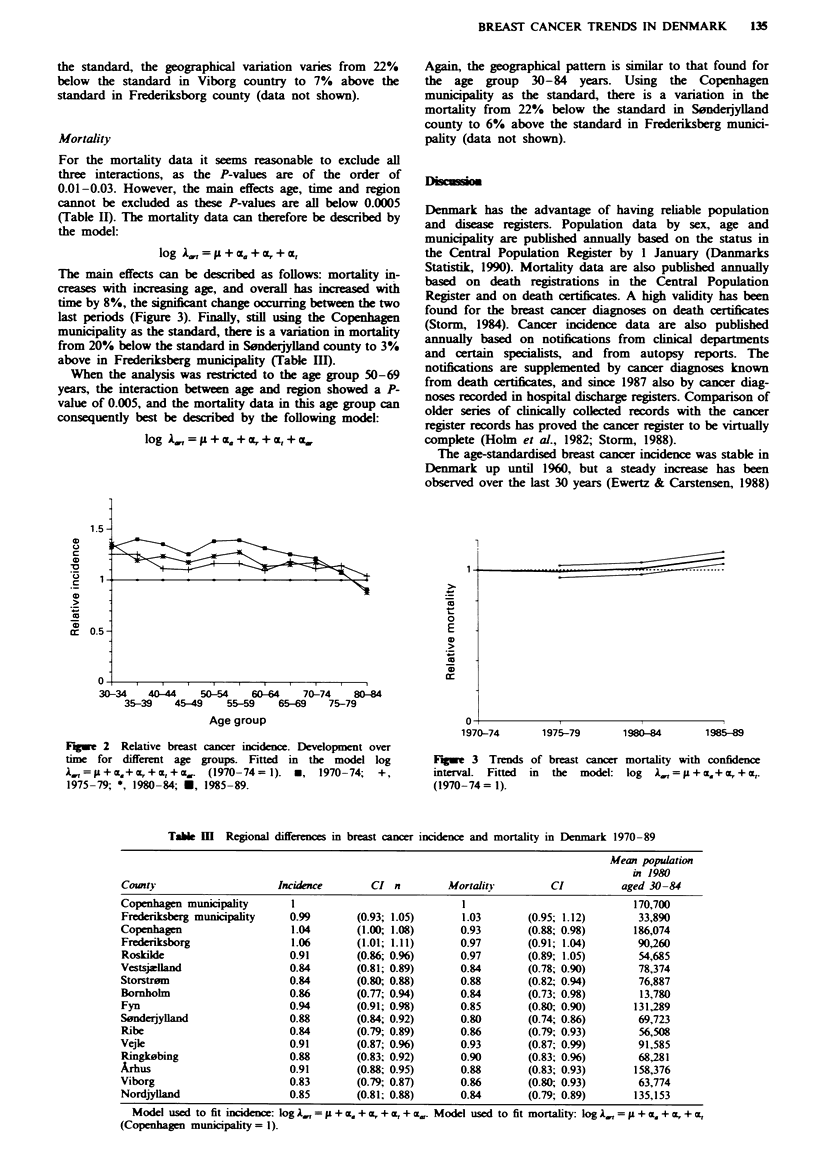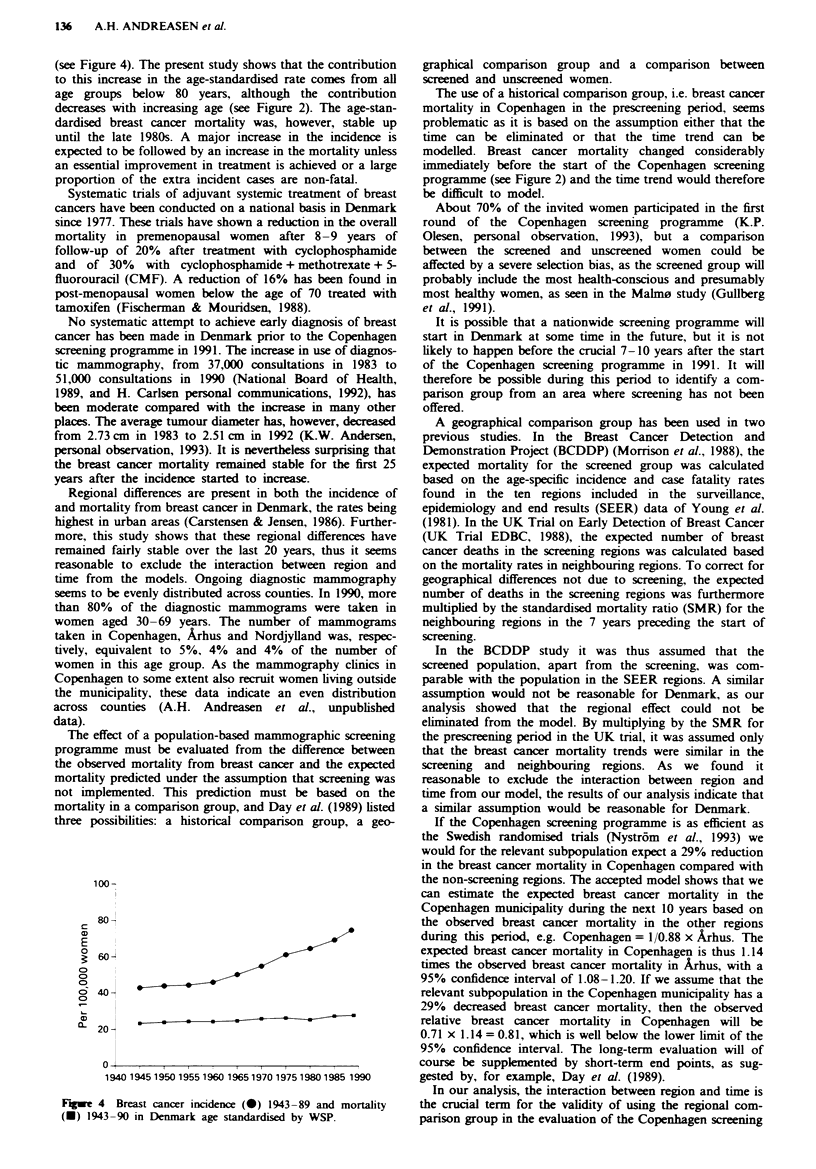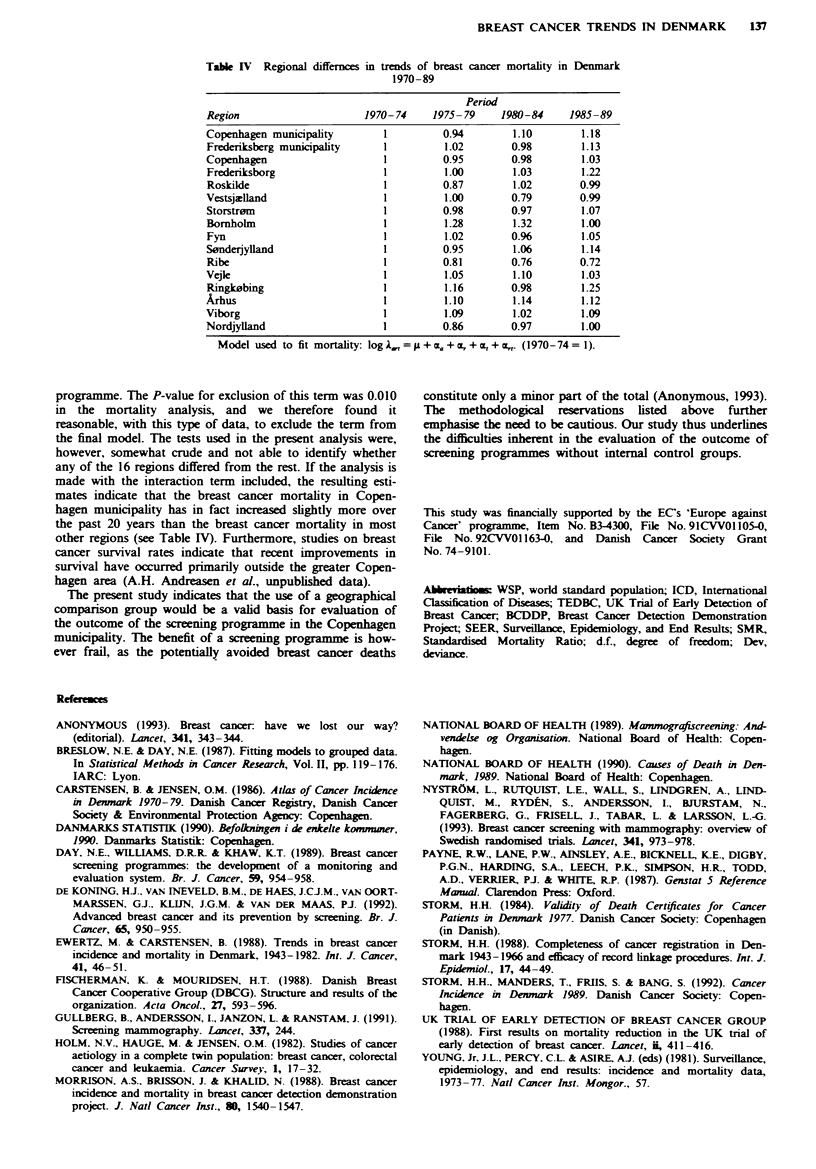# Regional trends in breast cancer incidence and mortality in Denmark prior to mammographic screening.

**DOI:** 10.1038/bjc.1994.262

**Published:** 1994-07

**Authors:** A. H. Andreasen, K. W. Andersen, M. Madsen, H. Mouridsen, K. P. Olesen, E. Lynge

**Affiliations:** Danish Cancer Society, Copenhagen, Denmark.

## Abstract

To provide a basis for the evaluation of mammographic screening programmes in Denmark, a study was undertaken of the regional differences in breast cancer incidence and mortality. All 16 regions were followed for the 20 year period, 1970-89, before the start of the first population-based mammographic screening programme in the Copenhagen municipality in 1991. Multiplicative Poisson models were used for the analysis. In general, the incidence increased during this period from 55 to 70 [per 100,000 standardised world standard population (WSP)], and the analysis shows this to be most pronounced among women below age 60. The mortality was more stable, changing only from 24 to 28 (per 100,000 standardised WSP), but a significant increase occurred in the late 1980s. The study showed regional differences in both incidence and mortality of breast cancer in Denmark. Both the incidence and the mortality varied between the regions, with maximum differences of 22%. The analysis showed no variation in the time trends in the different regions, and thus indicates that the use of a regional comparison group would be a valid basis for evaluation of the Copenhagen programme. Our study, however, underlies the difficulties inherent in the evaluation of screening programmes without internal control groups.


Br. J. Cancer (1994), 70, 133-137 © Macmillan Press Ltd., 1994

### Regional trends in breast cancer incidence and mortality in Denmark prior to mammographic screening

A.H. Andreasen[1], K.W. Andersen[2], M. Madsen[3], H. Mouridsen[2], K.P. Olesen[4] & E. Lynge[1]

[1]*Danish Cancer Society, Strandboulevarden 49, DK-2100 Copenhagen 0;* [2]*Danish Breast Cancer Cooperative Group, Rigshospitalet, Tagensvej 20, DK-2200 Copenhagen N;* [3]*Danish Institute for Clinical Epidemiology, Svanemøllevej 25, DK-2100 Copenhagen 0;* [4]*The Mammographic Clinic, Bispebjerg Hospital, Bispebjerg Bakke 23, DK-2400 Copenhagen NV, Denmark.*

**Summary** To provide a basis for the evaluation of mammographic screening programmes in Denmark, a study was undertaken of the regional differences in breast cancer incidence and mortality. All 16 regions were followed for the 20 year period, 1970-89, before the start of the first population-based mammographic screening programme in the Copenhagen municipality in 1991. Multiplicative Poisson models were used for the analysis. In general, the incidence increased during this period from 55 to 70 [per 100,000 standardised world standard population (WSP)], and the analysis shows this to be most pronounced among women below age 60. The mortality was more stable, changing only from 24 to 28 (per 100,000 standardised WSP), but a significant increase occurred in the late 1980s. The study showed regional differences in both incidence and mortality of breast cancer in Denmark. Both the incidence and the mortality varied between the regions, with maximum differences of 22%. The analysis showed no variation in the time trends in the different regions, and thus indicates that the use of a regional comparison group would be a valid basis for evaluation of the Copenhagen programme. Our study, however, underlies the difficulties inherent in the evaluation of screening programmes without internal control groups.

The first population-based mammographic screening programme started in the municipality of Copenhagen in April, 1991. All women in the municipality aged 50-69 years are invited every second year to a screening mammography. The programme comprises approximately 44,000 women, which is 8.3% of the total female population in this age group in Denmark. In 1993-94, at least three more of the 16 regions will start screening programmes.

The purpose of the screening is to detect and treat breast cancer cases earlier, and thus to achieve benefits for the patients through less radical treatment and prolonged life, as well as a reduction in effort and costs in the health care system (de Koning *et al.*, 1992). It is difficult to obtain an unbiased estimate of the prolonged life of the individual patient, and breast cancer mortality is therefore used as the most important measure of benefit for the patients. Day *et al.* (1989) emphasise the need to monitor the outcome of programmes to ensure that the aims of the programme are actually achieved.

The screening programme in Copenhagen is offered to all women aged 50-69 years, and no internal comparison group is thus available. One of the possibilities in this situation is to use regions without screening programmes as the control group. For such an evaluation to be valid it is necessary to assume that any changes in the breast cancer mortality would have been similar in the screening and the non-screening regions, provided no screening was introduced. The present study attempts to check this assumption by using data collected in the Danish population and disease registers. The paper describes the breast cancer incidence and mortality in Denmark during the 20 year period, 1970-89, before the first screening programme was started, and it discusses the possibility of using a regional comparison group for evaluating the potential reduction in breast cancer mortality. Data from before 1970 were disregarded as they were not considered relevant for evaluation of the screening activity in the 1990s.

### Materials and methods

### *Data*

Data on incident breast cancer cases from 1970-89 were retrieved from the files of the Danish Cancer Registry (Storm *et al.*, 1992). A total of 49,174 cases were identified from the ICD-7 codes 170. The data were tabulated by 5 year age groups (0-4, 5-9, ..., 85+) 5 year calendar periods (1970-74, 1975-79, 1980-84, 1985-89) and the 16 regions (Copenhagen municipality, Frederiksberg municipality and 14 counties) (see Figure 1).

Data on breast cancer deaths during 1970-89 were retrieved from the files of the Causes of Death Registry at the Danish Institute for Clinical Epidemiology (National Board of Health, 1990). In total, 22,891 deaths were identified from

**Figure 1** Denmark divided into counties. 1, Copenhagen municipality; 2, Frederiksberg municipality (not marked, geographically surrounded by the Copenhagen municipality); 3, Copenhagen; 4, Frederiksborg; 5, Roskilde; 6, Vestsjælland; 7, Storstrøm; 8, Bornholm; 9, Fyn; 10, Sønderjylland; 11, Ribe; 12, Vejle; 13, Ringkøbing; 14, Århus; 15, Viborg; and 16, Nordjylland counties.

Correspondence: E. Lynge.
Received 10 August 1993; and in revised form 4 January 1994.

the ICD-8 code 174 with breast cancer as the underlying cause of death. The deaths were tabulated in the same way as the incident cases. Table I shows the incident cases and deaths by age group and period.

Data on the female population during the period 1970–89 were retrieved from the files of the National Bureau of Statistics (Danmarks Statistik, 1990). These data were used to calculate the number of years lived at risk.

*Analysis*

The study of the geographical differences in incidence and mortality was separated into two analyses using equivalent multiplicative Poisson models (Breslow & Day, 1987, pp. 119–176). It is assumed that for any group specified by the variables age, time and county, the number of cases would be a random variable, independent of the others, and Poisson distributed with mean $\lambda_{art}n_{art}$. $\lambda_{art}$ represents the rate (incidence or mortality) and $n_{art}$ represents the number of years lived at risk in the group specified by age $= a$, region $= r$ and time $= t$. Given the short observation period, birth cohorts were not considered in the analysis. In both analyses the age groups 0–29 and 85 + were excluded, leaving 46,320 incident cases and 20,675 deaths, and the analyses are consequently based on 704 (= four time periods × 16 regions × 11 age groups) incidence rates and 704 mortality rates.

The analysis was repeated covering only the age group 50–69 years. These analyses thus cover 21,585 incident cases and 10,026 deaths, and are based on 256 (= four time periods × 16 regions × four age groups) rates.

The most complex statistical model we have studied includes all the main effects and all the two-factor interactions, i.e. in log-linear representation:

$$\log \lambda_{art} = \mu + \alpha_a + \alpha_r + \alpha_t + \alpha_{ar} + \alpha_{at} + \alpha_{rt}$$

or, expressed in another way:

age*region + age*time + region*time, where age*region represents both of the main effects and the first-order interaction.

In the analysis likelihood ratio tests are used. All the tests are successive tests, in which only one interaction or main effect is excluded at a time. Since most of the tests have a large number of degrees of freedom, a traditional test level of 5% would be too conservative (i.e. all tests would be significant). Instead, the individual *P*-values are discussed at the relevant points in the text. Genstat 5 (Payne *et al.*, 1987) was used to fit the models.

**Table I** Total number of breast cancer cases and breast cancer deaths in Denmark 1970–89 tabulated by age and calendar period

| | *Period* | | | |
|---|---|---|---|---|
| *Age group* | *1970–74* | *1975–79* | *1980–84* | *1985–89* |
| 0–4 | 0/0 | 0/0 | 0/0 | 0/0 |
| 5–9 | 0/0 | 0/0 | 0/0 | 0/0 |
| 10–14 | 0/0 | 0/0 | 1/1 | 0/0 |
| 15–19 | 1/0 | 0/0 | 1/1 | 0/0 |
| 20–24 | 9/2 | 8/5 | 4/1 | 8/1 |
| 25–29 | 51/12 | 67/18 | 61/7 | 41/7 |
| 30–34 | 131/51 | 205/57 | 212/49 | 196/40 |
| 35–39 | 326/107 | 453/82 | 537/115 | 603/112 |
| 40–44 | 655/190 | 737/207 | 902/191 | 1,228/266 |
| 45–49 | 1,094/355 | 1,123/314 | 1,209/289 | 1,421/383 |
| 50–54 | 1,122/514 | 1,270/495 | 1,238/477 | 1,356/504 |
| 55–59 | 1,139/638 | 1,340/603 | 1,417/607 | 1,432/638 |
| 60–64 | 1,264/656 | 1,354/709 | 1,424/648 | 1,585/724 |
| 65–69 | 1,175/638 | 1,494/667 | 1,420/705 | 1,555/803 |
| 70–74 | 1,101/654 | 1,353/690 | 1,524/841 | 1,553/769 |
| 75–79 | 910/551 | 1,188/614 | 1,261/724 | 1,334/819 |
| 80–84 | 695/406 | 884/495 | 872/595 | 1,028/683 |
| 85 + | 453/315 | 651/460 | 718/618 | 780/768 |
| Total | 10,126/5,089 | 12,127/5,416 | 12,801/5,869 | 14,120/6,517 |

**Results**

*Incidence*

Table II summarises the analysis of the multiplicative Poisson models covering the age group 30–84 years. In the incidence model the interactions between region and time (region*time) and age and region (age*region) can be excluded, as the *P*-values are of the order of 0.02. However, the interaction between age and time (age*time) and the main effect of region cannot be excluded, as both *P*-values are below 0.0005. Thus the incidence data can be described by the model:

$$\log \lambda_{art} = \mu + \alpha_a + \alpha_r + \alpha_t + \alpha_{at}$$

The age*time effect is shown in Figure 2, in which the incidence in the period 1970–74 is equal to 1 in each age group. The incidence in younger women (age 30–59) increases steadily, reaching almost 40% during the study period. This is combined with a smaller increase of 10–30% in incidence in elderly women (age 60–79), turning to a decrease of about 10% in the oldest age group (age 80–84). Using the Copenhagen municipality as the standard, the geographical variation varies from 17% below the standard in Viborg county to 6% above the standard in Frederisksborg county (Table III).

When the analysis was restricted to the age group 50–69 years, all the two-factor interactions could be excluded and the incidence data for this age group could consequently be described by the model:

$$\log \lambda_{art} = \mu + \alpha_a + \alpha_r + \alpha_t$$

The geographical pattern is similar to that found for the age group 30–84 years. Using the Copenhagen municipality as

**Table II** Breast cancer incidence and mortality in Denmark 1970–89 analysed by age, calendar period and region using multiplicative Poisson models

(a) Model fit

| | | | *Dev* | |
|---|---|---|---|---|
| | *Model* | *d.f.* | *Incidence* | *Mortality* |
| A | $a^*r + a^*t + r^*t$ | 450 | 465 | 427 |
| B1 | $a^*r + a^*t$ | 495 | 531 | 498 |
| B2 | $a^*r + r^*t$ | 480 | 576 | 473 |
| B3 | $a^*t + r^*t$ | 600 | 654 | 619 |
| C1 | $a^*r + t$ | 525 | 646 | 544 |
| C2 | $a^*t + r$ | 645 | 720 | 689 |
| C3 | $r^*t + a$ | 630 | 763 | 665 |
| D1 | $a^*r$ | 528 | 911 | 569 |
| D2 | $a^*t$ | 660 | 1,021 | 790 |
| D3 | $r^*t$ | 640 | 15,762 | 12,953 |
| D4 | $a + r + t$ | 675 | 833 | 735 |
| E1 | $a + r$ | 678 | 1,098 | 760 |
| E2 | $a + t$ | 690 | 1,133 | 836 |
| E3 | $r + t$ | 685 | 15,858 | 13,031 |
| F1 | $a$ | 693 | 1,394 | 859 |
| F2 | $r$ | 688 | 16,125 | 13,064 |
| F3 | $t$ | 700 | 16,397 | 13,495 |
| G | constant | 703 | 16,628 | 13,517 |

(b) Tests

| | *Test* | *Effect* | *d.f.* | *Dev* | *P* |
|---|---|---|---|---|---|
| *Incidence test* | | | | | |
| A | against B1 | $r^*t$ | 45 | 66 | 0.021 |
| B1 | against C2 | $a^*r$ | 150 | 189 | 0.017 |
| C2 | against D4 | $a^*t$ | 30 | 113 | <0.0005 |
| C2 | against D2 | $r$ | 15 | 301 | <0.0005 |
| *Mortality test* | | | | | |
| A | against B2 | $a^*t$ | 30 | 46 | 0.030 |
| B2 | against C3 | $a^*r$ | 150 | 192 | 0.012 |
| C3 | against D4 | $r^*t$ | 45 | 70 | 0.010 |
| D4 | against E3 | $a$ | 10 | 12,296 | <0.0005 |
| D4 | against E2 | $r$ | 15 | 101 | <0.0005 |
| D4 | against E1 | $t$ | 3 | 25 | <0.0005 |

d.f, degrees of freedom; Dev, deviance.

the standard, the geographical variation varies from 22% below the standard in Viborg country to 7% above the standard in Frederiksborg county (data not shown).

*Mortality*

For the mortality data it seems reasonable to exclude all three interactions, as the $P$-values are of the order of 0.01–0.03. However, the main effects age, time and region cannot be excluded as these $P$-values are all below 0.0005 (Table II). The mortality data can therefore be described by the model:

$$\log \lambda_{art} = \mu + \alpha_a + \alpha_r + \alpha_t$$

The main effects can be described as follows: mortality increases with increasing age, and overall has increased with time by 8%, the significant change occurring between the two last periods (Figure 3). Finally, still using the Copenhagen municipality as the standard, there is a variation in mortality from 20% below the standard in Sønderjylland county to 3% above in Frederiksberg municipality (Table III).

When the analysis was restricted to the age group 50–69 years, the interaction between age and region showed a $P$-value of 0.005, and the mortality data in this age group can consequently best be described by the following model:

$$\log \lambda_{art} = \mu + \alpha_a + \alpha_r + \alpha_t + \alpha_{ar}$$

Again, the geographical pattern is similar to that found for the age group 30–84 years. Using the Copenhagen municipality as the standard, there is a variation in the mortality from 22% below the standard in Sønderjylland county to 6% above the standard in Frederiksberg municipality (data not shown).

### Discussion

Denmark has the advantage of having reliable population and disease registers. Population data by sex, age and municipality are published annually based on the status in the Central Population Register by 1 January (Danmarks Statistik, 1990). Mortality data are also published annually based on death registrations in the Central Population Register and on death certificates. A high validity has been found for the breast cancer diagnoses on death certificates (Storm, 1984). Cancer incidence data are also published annually based on notifications from clinical departments and certain specialists, and from autopsy reports. The notifications are supplemented by cancer diagnoses known from death certificates, and since 1987 also by cancer diagnoses recorded in hospital discharge registers. Comparison of older series of clinically collected records with the cancer register records has proved the cancer register to be virtually complete (Holm *et al.*, 1982; Storm, 1988).

The age-standardised breast cancer incidence was stable in Denmark up until 1960, but a steady increase has been observed over the last 30 years (Ewertz & Carstensen, 1988)

**Figure 2** Relative breast cancer incidence. Development over time for different age groups. Fitted in the model $\log \lambda_{art} = \mu + \alpha_a + \alpha_r + \alpha_t + \alpha_{at}$. (1970–74 = 1). ■, 1970–74; +, 1975–79; *, 1980–84; ■, 1985–89.

**Figure 3** Trends of breast cancer mortality with confidence interval. Fitted in the model: $\log \lambda_{art} = \mu + \alpha_a + \alpha_r + \alpha_t$. (1970–74 = 1).

**Table III** Regional differences in breast cancer incidence and mortality in Denmark 1970–89

| *County* | *Incidence* | *CI n* | *Mortality* | *CI* | *Mean population in 1980 aged 30–84* |
|---|---|---|---|---|---|
| Copenhagen municipality | 1 | | 1 | | 170,700 |
| Frederiksberg municipality | 0.99 | (0.93; 1.05) | 1.03 | (0.95; 1.12) | 33,890 |
| Copenhagen | 1.04 | (1.00; 1.08) | 0.93 | (0.88; 0.98) | 186,074 |
| Frederiksborg | 1.06 | (1.01; 1.11) | 0.97 | (0.91; 1.04) | 90,260 |
| Roskilde | 0.91 | (0.86; 0.96) | 0.97 | (0.89; 1.05) | 54,685 |
| Vestsjælland | 0.84 | (0.81; 0.89) | 0.84 | (0.78; 0.90) | 78,374 |
| Storstrøm | 0.84 | (0.80; 0.88) | 0.88 | (0.82; 0.94) | 76,887 |
| Bornholm | 0.86 | (0.77; 0.94) | 0.84 | (0.73; 0.98) | 13,780 |
| Fyn | 0.94 | (0.91; 0.98) | 0.85 | (0.80; 0.90) | 131,289 |
| Sønderjylland | 0.88 | (0.84; 0.92) | 0.80 | (0.74; 0.86) | 69,723 |
| Ribe | 0.84 | (0.79; 0.89) | 0.86 | (0.79; 0.93) | 56,508 |
| Vejle | 0.91 | (0.87; 0.96) | 0.93 | (0.87; 0.99) | 91,585 |
| Ringkøbing | 0.88 | (0.83; 0.92) | 0.90 | (0.83; 0.96) | 68,281 |
| Århus | 0.91 | (0.88; 0.95) | 0.88 | (0.83; 0.93) | 158,376 |
| Viborg | 0.83 | (0.79; 0.87) | 0.86 | (0.80; 0.93) | 63,774 |
| Nordjylland | 0.85 | (0.81; 0.88) | 0.84 | (0.79; 0.89) | 135,153 |

Model used to fit incidence: $\log \lambda_{art} = \mu + \alpha_a + \alpha_r + \alpha_t + \alpha_{ar}$. Model used to fit mortality: $\log \lambda_{art} = \mu + \alpha_a + \alpha_r + \alpha_t$ (Copenhagen municipality = 1).

(see Figure 4). The present study shows that the contribution to this increase in the age-standardised rate comes from all age groups below 80 years, although the contribution decreases with increasing age (see Figure 2). The age-standardised breast cancer mortality was, however, stable up until the late 1980s. A major increase in the incidence is expected to be followed by an increase in the mortality unless an essential improvement in treatment is achieved or a large proportion of the extra incident cases are non-fatal.

Systematic trials of adjuvant systemic treatment of breast cancers have been conducted on a national basis in Denmark since 1977. These trials have shown a reduction in the overall mortality in premenopausal women after 8–9 years of follow-up of 20% after treatment with cyclophosphamide and of 30% with cyclophosphamide + methotrexate + 5-fluorouracil (CMF). A reduction of 16% has been found in post-menopausal women below the age of 70 treated with tamoxifen (Fischerman & Mouridsen, 1988).

No systematic attempt to achieve early diagnosis of breast cancer has been made in Denmark prior to the Copenhagen screening programme in 1991. The increase in use of diagnostic mammography, from 37,000 consultations in 1983 to 51,000 consultations in 1990 (National Board of Health, 1989, and H. Carlsen personal communications, 1992), has been moderate compared with the increase in many other places. The average tumour diameter has, however, decreased from 2.73 cm in 1983 to 2.51 cm in 1992 (K.W. Andersen, personal observation, 1993). It is nevertheless surprising that the breast cancer mortality remained stable for the first 25 years after the incidence started to increase.

Regional differences are present in both the incidence of and mortality from breast cancer in Denmark, the rates being highest in urban areas (Carstensen & Jensen, 1986). Furthermore, this study shows that these regional differences have remained fairly stable over the last 20 years, thus it seems reasonable to exclude the interaction between region and time from the models. Ongoing diagnostic mammography seems to be evenly distributed across counties. In 1990, more than 80% of the diagnostic mammograms were taken in women aged 30–69 years. The number of mammograms taken in Copenhagen, Århus and Nordjylland was, respectively, equivalent to 5%, 4% and 4% of the number of women in this age group. As the mammography clinics in Copenhagen to some extent also recruit women living outside the municipality, these data indicate an even distribution across counties (A.H. Andreasen et al., unpublished data).

The effect of a population-based mammographic screening programme must be evaluated from the difference between the observed mortality from breast cancer and the expected mortality predicted under the assumption that screening was not implemented. This prediction must be based on the mortality in a comparison group, and Day et al. (1989) listed three possibilities: a historical comparison group, a geographical comparison group and a comparison between screened and unscreened women.

**Figure 4** Breast cancer incidence (●) 1943–89 and mortality (■) 1943–90 in Denmark age standardised by WSP.

The use of a historical comparison group, i.e. breast cancer mortality in Copenhagen in the prescreening period, seems problematic as it is based on the assumption either that the time can be eliminated or that the time trend can be modelled. Breast cancer mortality changed considerably immediately before the start of the Copenhagen screening programme (see Figure 2) and the time trend would therefore be difficult to model.

About 70% of the invited women participated in the first round of the Copenhagen screening programme (K.P. Olesen, personal observation, 1993), but a comparison between the screened and unscreened women could be affected by a severe selection bias, as the screened group will probably include the most health-conscious and presumably most healthy women, as seen in the Malmø study (Gullberg et al., 1991).

It is possible that a nationwide screening programme will start in Denmark at some time in the future, but it is not likely to happen before the crucial 7–10 years after the start of the Copenhagen screening programme in 1991. It will therefore be possible during this period to identify a comparison group from an area where screening has not been offered.

A geographical comparison group has been used in two previous studies. In the Breast Cancer Detection and Demonstration Project (BCDDP) (Morrison et al., 1988), the expected mortality for the screened group was calculated based on the age-specific incidence and case fatality rates found in the ten regions included in the surveillance, epidemiology and end results (SEER) data of Young et al. (1981). In the UK Trial on Early Detection of Breast Cancer (UK Trial EDBC, 1988), the expected number of breast cancer deaths in the screening regions was calculated based on the mortality rates in neighbouring regions. To correct for geographical differences not due to screening, the expected number of deaths in the screening regions was furthermore multiplied by the standardised mortality ratio (SMR) for the neighbouring regions in the 7 years preceding the start of screening.

In the BCDDP study it was thus assumed that the screened population, apart from the screening, was comparable with the population in the SEER regions. A similar assumption would not be reasonable for Denmark, as our analysis showed that the regional effect could not be eliminated from the model. By multiplying by the SMR for the prescreening period in the UK trial, it was assumed only that the breast cancer mortality trends were similar in the screening and neighbouring regions. As we found it reasonable to exclude the interaction between region and time from our model, the results of our analysis indicate that a similar assumption would be reasonable for Denmark.

If the Copenhagen screening programme is as efficient as the Swedish randomised trials (Nyström et al., 1993) we would for the relevant subpopulation expect a 29% reduction in the breast cancer mortality in Copenhagen compared with the non-screening regions. The accepted model shows that we can estimate the expected breast cancer mortality in the Copenhagen municipality during the next 10 years based on the observed breast cancer mortality in the other regions during this period, e.g. Copenhagen = 1/0.88 × Århus. The expected breast cancer mortality in Copenhagen is thus 1.14 times the observed breast cancer mortality in Århus, with a 95% confidence interval of 1.08–1.20. If we assume that the relevant subpopulation in the Copenhagen municipality has a 29% decreased breast cancer mortality, then the observed relative breast cancer mortality in Copenhagen will be 0.71 × 1.14 = 0.81, which is well below the lower limit of the 95% confidence interval. The long-term evaluation will of course be supplemented by short-term end points, as suggested by, for example, Day et al. (1989).

In our analysis, the interaction between region and time is the crucial term for the validity of using the regional comparison group in the evaluation of the Copenhagen screening

**Table IV** Regional differnces in trends of breast cancer mortality in Denmark 1970–89

| *Region* | *Period* 1970–74 | 1975–79 | 1980–84 | 1985–89 |
|---|---|---|---|---|
| Copenhagen municipality | 1 | 0.94 | 1.10 | 1.18 |
| Frederiksberg municipality | 1 | 1.02 | 0.98 | 1.13 |
| Copenhagen | 1 | 0.95 | 0.98 | 1.03 |
| Frederiksborg | 1 | 1.00 | 1.03 | 1.22 |
| Roskilde | 1 | 0.87 | 1.02 | 0.99 |
| Vestsjælland | 1 | 1.00 | 0.79 | 0.99 |
| Storstrøm | 1 | 0.98 | 0.97 | 1.07 |
| Bornholm | 1 | 1.28 | 1.32 | 1.00 |
| Fyn | 1 | 1.02 | 0.96 | 1.05 |
| Sønderjylland | 1 | 0.95 | 1.06 | 1.14 |
| Ribe | 1 | 0.81 | 0.76 | 0.72 |
| Vejle | 1 | 1.05 | 1.10 | 1.03 |
| Ringkøbing | 1 | 1.16 | 0.98 | 1.25 |
| Århus | 1 | 1.10 | 1.14 | 1.12 |
| Viborg | 1 | 1.09 | 1.02 | 1.09 |
| Nordjylland | 1 | 0.86 | 0.97 | 1.00 |

Model used to fit mortality: $\log \lambda_{art} = \mu + \alpha_a + \alpha_r + \alpha_t + \alpha_{rt}$. (1970–74 = 1).

programme. The *P*-value for exclusion of this term was 0.010 in the mortality analysis, and we therefore found it reasonable, with this type of data, to exclude the term from the final model. The tests used in the present analysis were, however, somewhat crude and not able to identify whether any of the 16 regions differed from the rest. If the analysis is made with the interaction term included, the resulting estimates indicate that the breast cancer mortality in Copenhagen municipality has in fact increased slightly more over the past 20 years than the breast cancer mortality in most other regions (see Table IV). Furthermore, studies on breast cancer survival rates indicate that recent improvements in survival have occurred primarily outside the greater Copenhagen area (A.H. Andreasen *et al.*, unpublished data).

The present study indicates that the use of a geographical comparison group would be a valid basis for evaluation of the outcome of the screening programme in the Copenhagen municipality. The benefit of a screening programme is however frail, as the potentially avoided breast cancer deaths constitute only a minor part of the total (Anonymous, 1993). The methodological reservations listed above further emphasise the need to be cautious. Our study thus underlines the difficulties inherent in the evaluation of the outcome of screening programmes without internal control groups.

This study was financially supported by the EC's 'Europe against Cancer' programme, Item No. B3-4300, File No. 91CVV01105-0, File No. 92CVV01163-0, and Danish Cancer Society Grant No. 74-9101.

**Abbreviations:** WSP, world standard population; ICD, International Classification of Diseases; TEDBC, UK Trial of Early Detection of Breast Cancer; BCDDP, Breast Cancer Detection Demonstration Project; SEER, Surveillance, Epidemiology, and End Results; SMR, Standardised Mortality Ratio; d.f., degree of freedom; Dev, deviance.

### References

ANONYMOUS (1993). Breast cancer: have we lost our way? (editorial). *Lancet*, **341**, 343–344.

BRESLOW, N.E. & DAY, N.E. (1987). Fitting models to grouped data. In *Statistical Methods in Cancer Research*, Vol. II, pp. 119–176. IARC: Lyon.

CARSTENSEN, B. & JENSEN, O.M. (1986). *Atlas of Cancer Incidence in Denmark 1970–79*. Danish Cancer Registry, Danish Cancer Society & Environmental Protection Agency: Copenhagen.

DANMARKS STATISTIK (1990). *Befolkningen i de enkelte kommuner, 1990*. Danmarks Statistik: Copenhagen.

DAY, N.E., WILLIAMS, D.R.R. & KHAW, K.T. (1989). Breast cancer screening programmes: the development of a monitoring and evaluation system. *Br. J. Cancer*, **59**, 954–958.

DE KONING, H.J., VAN INEVELD, B.M., DE HAES, J.C.J.M., VAN OORTMARSSEN, G.J., KLIJN, J.G.M. & VAN DER MAAS, P.J. (1992). Advanced breast cancer and its prevention by screening. *Br. J. Cancer*, **65**, 950–955.

EWERTZ, M. & CARSTENSEN, B. (1988). Trends in breast cancer incidence and mortality in Denmark, 1943–1982. *Int. J. Cancer*, **41**, 46–51.

FISCHERMAN, K. & MOURIDSEN, H.T. (1988). Danish Breast Cancer Cooperative Group (DBCG). Structure and results of the organization. *Acta Oncol.*, **27**, 593–596.

GULLBERG, B., ANDERSSON, I., JANZON, L. & RANSTAM, J. (1991). Screening mammography. *Lancet*, **337**, 244.

HOLM, N.V., HAUGE, M. & JENSEN, O.M. (1982). Studies of cancer aetiology in a complete twin population: breast cancer, colorectal cancer and leukaemia. *Cancer Survey*, **1**, 17–32.

MORRISON, A.S., BRISSON, J. & KHALID, N. (1988). Breast cancer incidence and mortality in breast cancer detection demonstration project. *J. Natl Cancer Inst.*, **80**, 1540–1547.

NATIONAL BOARD OF HEALTH (1989). *Mammografiscreening: Andvendelse og Organisation*. National Board of Health: Copenhagen.

NATIONAL BOARD OF HEALTH (1990). *Causes of Death in Denmark, 1989*. National Board of Health: Copenhagen.

NYSTRÖM, L., RUTQUIST, L.E., WALL, S., LINDGREN, A., LINDQUIST, M., RYDÉN, S., ANDERSSON, I., BJURSTAM, N., FAGERBERG, G., FRISELL, J., TABAR, L. & LARSSON, L.-G. (1993). Breast cancer screening with mammography: overview of Swedish randomised trials. *Lancet*, **341**, 973–978.

PAYNE, R.W., LANE, P.W., AINSLEY, A.E., BICKNELL, K.E., DIGBY, P.G.N., HARDING, S.A., LEECH, P.K., SIMPSON, H.R., TODD, A.D., VERRIER, P.J. & WHITE, R.P. (1987). *Genstat 5 Reference Manual*. Clarendon Press: Oxford.

STORM, H.H. (1984). *Validity of Death Certificates for Cancer Patients in Denmark 1977*. Danish Cancer Society: Copenhagen (in Danish).

STORM, H.H. (1988). Completeness of cancer registration in Denmark 1943–1966 and efficacy of record linkage procedures. *Int. J. Epidemiol.*, **17**, 44–49.

STORM, H.H., MANDERS, T., FRIIS, S. & BANG, S. (1992). *Cancer Incidence in Denmark 1989*. Danish Cancer Society: Copenhagen.

UK TRIAL OF EARLY DETECTION OF BREAST CANCER GROUP (1988). First results on mortality reduction in the UK trial of early detection of breast cancer. *Lancet*, **ii**, 411–416.

YOUNG, Jr, J.L., PERCY, C.L. & ASIRE, A.J. (eds) (1981). Surveillance, epidemiology, and end results: incidence and mortality data, 1973–77. *Natl Cancer Inst. Mongor.*, 57.